# Real-world clinical determinants of alcohol dependence in outpatients with bipolar disorder: a multicenter treatment survey for bipolar disorder in psychiatric outpatient clinics with 2,392 participants

**DOI:** 10.3389/fpsyt.2024.1434810

**Published:** 2024-11-07

**Authors:** Keita Tokumitsu, Norio Sugawara, Naoto Adachi, Yukihisa Kubota, Yoichiro Watanabe, Kazuhira Miki, Takaharu Azekawa, Koji Edagawa, Eiichi Katsumoto, Seiji Hongo, Eiichiro Goto, Hitoshi Ueda, Masaki Kato, Reiji Yoshimura, Atsuo Nakagawa, Toshiaki Kikuchi, Takashi Tsuboi, Koichiro Watanabe, Norio Yasui-Furukori

**Affiliations:** ^1^ Department of Psychiatry, Dokkyo Medical University School of Medicine, Tochigi, Japan; ^2^ The Japanese Association of Neuro-Psychiatric Clinics, Tokyo, Japan; ^3^ The Japanese Society of Clinical Neuropsychopharmacology, Tokyo, Japan; ^4^ Department of Neuropsychiatry, Kansai Medical University, Osaka, Japan; ^5^ Department of Psychiatry, University of Occupational and Environmental Health, Fukuoka, Japan; ^6^ Department of Neuropsychiatry, St. Marianna University School of Medicine, Kanagawa, Japan; ^7^ Department of Neuropsychiatry, Keio University School of Medicine, Tokyo, Japan; ^8^ Department of Neuropsychiatry, Kyorin University School of Medicine, Tokyo, Japan

**Keywords:** alcohol dependence, bipolar disorder, suicidal ideation, real-world, Japanese

## Abstract

**Background:**

Bipolar disorder is a psychiatric disorder characterized by mood swings between manic and depressed states that causes psychosocial problems. Cognitive function deteriorates with each recurrence, making it important to maintain remission through continued treatment. Bipolar disorder often co-occurs with alcohol dependence, which is known to lead to decreased treatment adherence and increased suicide risk. However, the real-world clinical determinants of alcohol dependence in outpatients with bipolar disorder in Japan remain unclear.

**Methods:**

We conducted an observational study targeting 2392 patients with bipolar disorder using data from the MUSUBI study, a joint project of the Japanese Association of Neuro-Psychiatric Clinics and the Japanese Society of Clinical Neuropsychopharmacology. After determining the prevalence of alcohol dependence and the sociodemographic characteristics of patients with bipolar disorder, multivariate analysis was performed to identify risk factors for alcohol comorbidity.

**Results:**

The prevalence of alcohol dependence among outpatients with bipolar disorder in this study was 5.7%. The prevalence was 7.6% for males and 3.1% for females. The results of the binomial logistic regression analysis revealed that bipolar I disorder, manic state, comorbidities with other psychiatric disorders, male sex, and suicidal ideation were significantly associated with alcohol dependence. Stratified analysis by gender showed that alcohol dependence was more strongly associated with the presence of suicidal ideation in women than in men.

**Limitation:**

First, because this was an observational study with a cross-sectional design, causal relationships between factors cannot be determined. In addition, this study included outpatients in Japan but lacked information on inpatients. Therefore, it was considered necessary to conduct the study on a larger population in order to generate more robust evidence.

**Conclusions:**

We found that outpatients with bipolar disorder, especially men, had higher rates of alcohol dependence overall than the general population in Japan. In addition, the relationship between alcohol dependence and suicidal ideation was stronger in women than in men with bipolar disorder. There was a strong association between manic states and alcohol dependence in outpatients with bipolar disorder. These results are useful to clinicians because they reinforce real-world clinical evidence for the treatment of bipolar disorder and co-occurring alcohol dependence.

## Introduction

Bipolar disorder is a psychiatric disorder characterized by mood swings between manic and depressed states that significantly negatively impact cognitive and social functioning (1). The estimated lifetime incidence is 0.6% for bipolar I disorder and 0.4% for bipolar II disorder (2). The average age of onset is 18 years for bipolar I disorder and 20 years for bipolar II disorder, with nearly equal prevalence among males and females (2). Notably, relatives of adults with bipolar I disorder have a tenfold risk of onset, indicating a genetic influence on the disorder (3). Furthermore, individuals with bipolar disorder have a 20-30 times greater risk of suicide than the general population (4). Additionally, the co-occurrence of alcohol problems in outpatients with bipolar disorder has been noted to increase suicidal behavior (5). Therefore, it is crucial to provide support for alcohol dependence for outpatients with bipolar disorder while paying attention to suicidal ideation. Given that functional impairment worsens with each recurrence of bipolar disorder, maintaining remission of mood swings through appropriate psychotherapy and pharmacotherapy to achieve functional recovery is important (6). However, previous studies have reported that comorbid substance use disorders reduce treatment adherence in patients with bipolar disorder and adversely affect patients’ prognosis (7). According to the latest WHO report released on June 25, 2024, approximately 400 million people, or about 7% of the global population aged 15 and over, suffer from alcohol use disorders, with an estimated 209 million people being alcohol dependent (8). The disability-adjusted life years (DALYs) due to alcohol use are estimated to be 2.3% for women and 8.9% for men, making it the 7th leading risk factor globally (9).

Alcohol is a frequent cause of substance abuse also in Japan, with an estimated 12-month incidence of alcohol dependence reaching 0.9% (10). Furthermore, women are thought to exhibit more severe adverse effects (e.g., cognitive and motor function) with relatively low doses of alcohol than men (11). Despite efforts such as the Healthy Japan 21 project, which aims to improve lifestyles and reduce alcohol consumption, the percentage of women who are habitual drinkers has increased significantly (12). Thus, the relationship between alcohol problems and bipolar disorder has significant public health implications in Japan. However, the factors associated with bipolar disorder and alcohol dependence in Japan, including sex differences, remain unclear.

A previous study reported that the risk factors for the comorbidity of bipolar disorder and alcohol use disorder in Australia included male sex, youth, and the presence of self-harming behavior (13). Nevertheless, there were several limitations to that study, such as data robustness issues due to small sample sizes, selection bias from a single-center study, and the lack of comparisons of bipolar subtypes (13). Therefore, we attempted to update the existing evidence on factors associated with the comorbidity of bipolar disorder and alcohol dependence with a particular focus on suicidal ideation and gender differences by performing multivariate analyses using a large-scale multicenter dataset.

In Japan, more than 90% of individuals diagnosed with mood disorders receive treatment as outpatients, and approximately half of them is treated at clinics affiliated with the Japanese Association of Neuro-Psychiatric Clinics (JAPC) (14). In this context, collaborative research, known as the MUlticenter treatment SUrvey on BIpolar disorder in Japanese psychiatric clinics (MUSUBI), was conducted by the JAPC and the Japanese Society of Clinical Neuropsychopharmacology (14–18). The aim of the project was to gather evidence for the real-world practical treatment of bipolar disorder in Japan. In this study, we aimed to investigate the prevalence of alcohol dependence among outpatients with bipolar disorder and identify risk factors for alcohol dependence in patients with bipolar disorder using data from the MUSUBI study.

## Subjects and methods

### Study design and subjects

In the MUSUBI study, a questionnaire was administered at 176 outpatient clinics belonging to the JAPC from September to October 2016. We collected data on outpatients with bipolar disorder who visited each psychiatric clinic from October 1, 2016, to December 31, 2016. The analysis included the baseline data for each patient at the time of the first visit during the study period. The classification of the bipolar subtype was determined one year after the observation baseline to September-October 2017. At each clinic, data were collected for up to 20 patients with bipolar disorder in the order that they visited the clinic (17). Participants were diagnosed with bipolar disorder and alcohol dependence based on the ICD-10 criteria (19). In addition, we classified bipolar I disorder and bipolar II disorder according to the DSM-5 criteria (20).

### Study procedures

Clinical psychiatrists were requested to participate in a retrospective medical record survey by filling out a questionnaire on outpatients with bipolar disorder. The questionnaire included the following participant characteristics: age at study entry, age at onset, sex, body mass index (BMI), bipolar subtypes, work status, educational background, mood status, intelligence quotient (IQ), Global Assessment of Functioning (GAF) score, mood stabilizer prescriptions, antidepressant prescriptions, antipsychotic prescriptions, anxiolytic prescriptions, hypnotic prescriptions, rapid cycling, other psychiatric comorbidities, physical comorbidities, psychotic symptoms, suicidal ideation and alcohol dependence.

### Statistical analysis

We conducted all statistical analyses using EZR (Saitama Medical Center, Jichi Medical University, Saitama, Japan) (21), which is a user-friendly interface for R (The R Foundation for Statistical Computing, Vienna, Austria, version 4.3.0). EZR is a customized version of R Commander (version 2.7-1) that integrates commonly used statistical functions in biostatistics.

We set the two-sided significance level for all statistical tests at 0.05 utilizing the chi-square test and the Mann−Whitney U test to examine differences in demographic and clinical characteristics between patients with and without alcohol dependence. Univariate analyses were conducted to identify demographic and clinical features. To thoroughly explore factors associated with alcohol dependence among patients with bipolar disorder, we employed binomial logistic regression with forced entry to ensure that no potential associations were overlooked. The independent variables included age at study entry, age at onset, sex, BMI, bipolar subtypes, work status, educational background, mood status, IQ, GAF score, mood stabilizer prescription, antidepressant prescriptions, antipsychotic prescriptions, anxiolytic prescriptions, hypnotic prescriptions, rapid cycling, other psychiatric comorbidities, physical comorbidities, psychotic symptoms and suicidal ideation.

### Ethics

This research was conducted following the Declaration of Helsinki and the Japanese Ethical Guidelines for Medical and Health Research Involving Human Subjects. Approval for the study protocol was obtained from the institutional review board of the ethics committee of the JAPC and Dokkyo Medical University School of Medicine before the research began. Because this study involved a retrospective examination of medical records, informed consent was waived. Nevertheless, we provided information about the research to allow patients the option to opt out when they wished (17). Our team obtained the necessary administrative permissions and licenses to access the data utilized in this study. The ethics committee of the Japanese Association of Neuro-Psychiatric Clinics set restrictions on data sharing because of potentially identifying or sensitive patient information. Please contact the institutional review board of the ethics committee when requesting data. Contact information for our ethics committee is as follows: The Institutional Review Board of the Ethics Committee of the Japanese Association of Neuro-Psychiatric Clinics; Shibuya-ku, Yoyogi 1-38-2, Tokyo Metropolis, Japan, Postal Code 151–0053, Phone +81-3-3320-1423.

## Results

We obtained questionnaire data for 2392 outpatients with bipolar disorder from 176 psychiatric clinics affiliated with the JAPC. The proportion of study participants with alcohol dependence was 5.2% (125/2392). Of these, 7.6% (85/1096) were male and 3.1% (40/1296) were female. The results of the univariate analysis are shown in **Table 1**. Because of multiple 20 comparisons, a Bonferroni correction was applied that yielded a corrected significance criterion of p <0.0025. According to the univariate analysis of alcohol dependence in bipolar patients, the bipolar subtypes, GAF score, mood status, physical comorbidities, psychotic symptoms, other psychiatric comorbidities, sex and the presence of suicidal ideation were significantly associated with alcohol dependence.

**Table 1:** Demographics and clinical characteristics for participants.

Factor	Group	Alcohol dependence (-)	Alcohol dependence (+)	p value
		n = 2267	n = 125	
Sex (%)	men: n (%)	1011 (44.6)	85 (68.0)	<0.001
	women: n (%)	1256 (55.4)	40 (32.0)	
Age at baseline [mean (SD)]		50.35 (13.63)	51.91 (12.05)	0.211
Age at onset [mean (SD)]		34.71 (12.40)	34.87 (10.80)	0.887
BMI [mean (SD)]		23.37 (3.82)	24.21 (3.97)	0.018
Bipolar subtypes	Type II: n (%)	1386 (61.1)	51 (40.8)	<0.001
	Type I: n (%)	734 (32.4)	63 (50.4)	
	Unclassifiable	147 ( 6.5)	11 ( 8.8)	
Mood status	Remission: n (%)	980 (43.2)	26 (20.8)	<0.001
	Depressive state: n (%)	903 (39.8)	54 (43.2)	
	Manic/hypomanic state: n (%)	180 ( 7.9)	16 (12.8)	
	Mixed feature: n (%)	204 ( 9.0)	29 (23.2)	
Rapid cycler	No: n (%)	2021 (89.1)	103 (82.4)	0.029
	Yes: n (%)	246 (10.9)	22 (17.6)	
Psychotic symptoms	No: n (%)	2099 (92.6)	105 (84.0)	0.001
	Yes: n (%)	168 ( 7.4)	20 (16.0)	
Suicidal ideation	No: n (%)	2045 (90.2)	91 (72.8)	<0.001
	Yes: n (%)	222 ( 9.8)	34 (27.2)	
Work status	Unemployed: n (%)	737 (32.5)	52 (41.6)	0.045
	Employed: n (%)	1530 (67.5)	73 (58.4)	
Educational background	Special support educational school: n (%)	7 ( 0.3)	2 ( 1.6)	0.132
	Junior high school: n (%)	109 ( 4.8)	5 ( 4.0)	
	High school or vocational school: n (%)	1063 (46.9)	53 (42.4)	
	Junior college or technical college: n (%)	211 ( 9.3)	8 ( 6.4)	
	University: n (%)	801 (35.3)	52 (41.6)	
	Master’s degree or higher: n (%)	76 ( 3.4)	5 ( 4.0)	
Intelligence quotient (IQ)	<70: n (%)	22 ( 1.0)	1 ( 0.8)	0.208
	>85: n (%)	2168 (95.6)	116 (92.8)	
	85-71: n (%)	77 ( 3.4)	8 ( 6.4)	
Mood stabilizer prescription	No: n (%)	370 (16.3)	18 (14.4)	0.658
	Yes: n (%)	1897 (83.7)	107 (85.6)	
Antipsychotics prescription	No: n (%)	1089 (48.0)	56 (44.8)	0.540
	Yes: n (%)	1178 (52.0)	69 (55.2)	
Antidepressants prescription	No: n (%)	1320 (58.2)	72 (57.6)	0.964
	Yes: n (%)	947 (41.8)	53 (42.4)	
Anxiolytics prescription	No: n (%)	1443 (63.7)	73 (58.4)	0.275
	Yes: n (%)	824 (36.3)	52 (41.6)	
Hypnotics prescription	No: n (%)	921 (40.6)	41 (32.8)	0.100
	Yes: n (%)	1346 (59.4)	84 (67.2)	
GAF score	81-100: n (%)	763 (33.7)	20 (16.0)	<0.001
	61-80: n (%)	1048 (46.2)	62 (49.6)	
	41-60: n (%)	402 (17.7)	38 (30.4)	
	1-40: n (%)	54 ( 2.4)	5 ( 4.0)	
Physical comorbidity	No: n (%)	1583 (69.8)	70 (56.0)	0.002
	Yes: n (%)	684 (30.2)	55 (44.0)	
Other psychiatric comorbidity	No: n (%)	1857 (81.9)	82 (65.6)	<0.001
	Yes: n (%)	410 (18.1)	43 (34.4)	

The results of the binomial logistic regression analysis for all participants are shown in **Table 2**. Our study revealed that outpatients with alcohol dependence and bipolar disorder had a significantly greater proportion of bipolar I disorder (odds ratio [OR] [95% CI] = 1.98 [1.31-2.98], p=0.001; reference factor = bipolar II disorder), manic/hypomanic state (OR = 3.06 [1.52-6.15], p=0.002; reference factor = remission), mixed feature state (OR = 3.46 [1.74-6.89], p<0.001; reference factor = remission), male sex (OR=3.40 [2.20-5.24], p<0.001), other psychiatric comorbidities (OR = 1.89 [1.20-2.98], p=0.006) and suicidal ideation (OR=2.65 [1.59-4.40], p<0.001).

**Table 2:** Binomial logistic regression analysis of factors associated alcohol dependence among all participants.

Factor	Odds ratio (95% confidence interval)	p value
(Intercept)	0.00 (0.00-0.01)	<0.001
Sex (being men)	3.40 (2.20-5.24)	<0.001
Age at baseline	1.01 (0.99-1.04)	0.160
Age at onset	1.00 (0.98-1.02)	0.770
BMI	1.01 (0.96-1.06)	0.830
Bipolar subtypes (reference; Type II)	reference	
Type I	1.98 (1.31-2.98)	0.001
Unclassifiable	1.46 (0.70-3.03)	0.310
Mood status (reference; Remission)	reference	
Depressive state	1.78 (0.99-3.22)	0.056
Manic/hypomanic state	3.06 (1.52-6.15)	0.002
Mixed feature	3.46 (1.74-6.89)	<0.001
Rapid cycler	1.23 (0.72-2.11)	0.450
Psychotic symptoms	1.26 (0.71-2.25)	0.440
Suicidal ideation	2.65 (1.59-4.40)	<0.001
Work status	1.08 (0.70-1.67)	0.730
Educational background (reference; Junior college, technical college, or higher)	reference	
High school, vocational school	0.91 (0.60-1.37)	0.660
Special support education school, junior high school	0.75 (0.30-1.88)	0.540
IQ>85	1.30 (0.59-2.90)	0.520
Mood stabilizer prescription	1.02 (0.59-1.75)	0.960
Antipsychotics prescription	0.80 (0.54-1.20)	0.290
Antidepressants prescription	0.95 (0.63-1.44)	0.820
Anxiolytics prescription	1.20 (0.81-1.80)	0.360
Hypnotics prescription	1.11 (0.73-1.67)	0.620
GAF score (reference; 81-100)	reference	
61-80	1.34 (0.73-2.46)	0.350
1-60	1.34 (0.63-2.85)	0.440
Physical.comorbidity	1.41 (0.95-2.11)	0.091
Other psychiatric comorbidity	1.89 (1.20-2.98)	0.006

Next, we present the results of the multivariate analysis stratified by sex. The results of the binomial logistic regression analysis of factors associated with alcohol dependence among male participants are shown in **Table 3**. Male patients with alcohol dependence were significantly older at baseline, had a greater proportion of bipolar I disorder (OR= 2.25 [1.35-3.75], p=0.002; reference factor = bipolar II disorder), were in a depressive state (OR= 2.29 [1.13-4.64], p=0.021; reference factor = remission), were in a manic/hypomanic state (OR= 2.71 [1.14-6.43], p=0.023; reference factor = remission), were in a mixed feature state (OR= 2.78 [1.11-6.95], p=0.028; reference factor = remission), had other psychiatric comorbidities (OR = 1.95 [1.08-3.52], p=0.027) and had suicidal ideation (OR=2.13 [1.07-4.24], p=0.030).

**Table 3:** Binomial logistic regression analysis of factors associated alcohol dependence among male participants.

Factor	Odds ratio (95% confidence interval)	p value
(Intercept)	0.00 (0.00-0.02)	<0.001
Age at baseline	1.03 (1.00-1.06)	0.021
Age at onset	1.00 (0.97-1.02)	0.7
BMI	1.03 (0.96-1.10)	0.42
Bipolar subtypes (reference; Type II)	reference	
Type I	2.25 (1.35-3.75)	0.002
Unclassifiable	2.22 (0.93-5.29)	0.072
Mood status (reference; Remission)	reference	
Depressive state	2.29 (1.13-4.64)	0.021
Manic/hypomanic state	2.71 (1.14-6.43)	0.023
Mixed feature	2.78 (1.11-6.95)	0.028
Rapid cycler	1.63 (0.78-3.39)	0.19
Psychotic symptoms	0.62 (0.25-1.53)	0.3
Suicidal ideation	2.13 (1.07-4.24)	0.03
Work status	1.47 (0.83-2.59)	0.18
Educational background (reference; Junior college, technical college, or higher)	reference	
High school, vocational school	0.88 (0.52-1.48)	0.62
Special support education school, junior high school	1.30 (0.43-3.92)	0.64
IQ>85	0.69 (0.19-2.52)	0.57
Mood stabilizer prescription	1.09 (0.55-2.16)	0.8
Antipsychotics prescription	0.72 (0.43-1.19)	0.2
Antidepressants prescription	1.19 (0.71-1.99)	0.51
Anxiolytics prescription	1.08 (0.65-1.80)	0.78
Hypnotics prescription	0.89 (0.55-1.46)	0.66
GAF score (reference; 81-100)	reference	
61-80	1.47 (0.71-3.04)	0.3
1-60	1.86 (0.75-4.66)	0.18
Physical.comorbidity	1.55 (0.95-2.54)	0.077
Other psychiatric comorbidity	1.95 (1.08-3.52)	0.027

The results of the binomial logistic regression analysis of factors associated with alcohol dependence among female participants are shown in **Table 4**. Female patients with alcohol dependence had significant psychotic symptoms (OR=3.33 [1.45-7.64], p=0.005) and suicidal ideation (OR=2.93 [1.27-6.77], p=0.012). Suicidal ideation was found to be a factor associated with alcohol dependence in both genders, with a stronger correlation observed in women than in men. (odds ratio: 2.13 for men and 2.93 for women).

**Table 4:** Binomial logistic regression analysis of factors associated alcohol dependence among female participants.

Factor	Odds ratio (95% confidence interval)	p value
(Intercept)	0.01 (0.00-0.14)	<0.001
Age at baseline	0.99 (0.96-1.03)	0.760
Age at onset	1.01 (0.97-1.05)	0.750
BMI	0.98 (0.90-1.07)	0.700
Bipolar subtypes (reference; Type II)	reference	
Type I	1.35 (0.63-2.89)	0.440
Unclassifiable	0.60 (0.12-2.87)	0.520
Mood status (reference; Remission)	reference	
Depressive state	0.96 (0.29-3.11)	0.940
Manic/hypomanic state	3.09 (0.88-10.90)	0.080
Mixed feature	3.24 (0.98-10.70)	0.054
Rapid cycler	0.93 (0.39-2.22)	0.870
Psychotic symptoms	3.33 (1.45-7.64)	0.005
Suicidal ideation	2.93 (1.27-6.77)	0.012
Work status	0.88 (0.41-1.90)	0.750
Educational background (reference; Junior college, technical college, or higher)	reference	
High school, vocational school	1.05 (0.50-2.23)	0.890
Special support education school, junior high school	0.33 (0.05-2.02)	0.230
IQ>85	2.47 (0.82-7.47)	0.110
Mood stabilizer prescription	1.16 (0.44-3.04)	0.760
Antipsychotics prescription	1.13 (0.55-2.31)	0.750
Antidepressants prescription	0.61 (0.29-1.30)	0.200
Anxiolytics prescription	1.47 (0.72-3.02)	0.290
Hypnotics prescription	1.87 (0.81-4.33)	0.140
GAF score (reference; 81-100)	reference	
61-80	1.11 (0.35-3.53)	0.860
1-60	0.80 (0.20-3.30)	0.760
Physical.comorbidity	1.22 (0.57-2.63)	0.610
Other psychiatric comorbidity	1.90 (0.87-4.13)	0.100

## Discussion

In this study, we found that the prevalence of alcohol dependence among patients with bipolar disorder was 5.2%. This is notably higher than the prevalence of alcohol dependence in the general population (22). A significant result of this study was the identification of suicidal ideation as an important factor associated with alcohol dependence in outpatients with bipolar disorder. This finding remained consistent even when stratified by sex. Previous research has shown that the co-occurrence of alcohol use disorders in individuals with bipolar disorder increases the rate of suicide attempts (23). The lifetime suicide attempt rate in bipolar disorder patients with comorbid substance use disorders is reported to be 39.5%, compared to 23.8% in those without comorbidities (24). Furthermore, bipolar disorder patients with comorbid substance use disorders have a higher mortality rate, with alcohol problems particularly affecting this increase in the mortality rate (25). In fact, 16.5% of men and 14.4% of women with bipolar disorder and comorbid alcohol substance use disorders have been reported to have completed suicide (26). Regarding the prevalence of suicide, the presence of alcohol use disorder in individuals with bipolar disorder increases the suicide completion rate by 1.46 times in men and 3.11 times in women; this is a particularly noticeable effect on the increase in suicide completion among women (26). Our study revealed that women with comorbid alcohol dependence and bipolar disorder had greater odds ratios for suicidal ideation than men did (suicidal ideation associated with alcohol dependence; male participants OR = 2.13, female participants OR = 2.93). Targeted interventions that address gender differences in alcohol dependence could prevent suicide in patients with bipolar disorder.

The association between sex differences and alcohol dependence is an important issue not only in bipolar patients but also in the general population. Previous research has shown that in Asian countries, the co-occurrence of substance use disorders is more common in men (27). However, the proportion of young women with drinking habits in Japan is increasing annually (12). The Japanese government has explicitly addressed alcohol issues and emphasized the health risks associated with drinking as part of its social policy called “Health Japan 21” (12). Therefore, it is important to raise awareness of these issues throughout society. Previous studies have reported that the prevalence of current alcohol dependence in Japan is 1.0% in men and 0.1% in women, indicating a gender difference of approximately tenfold (28). However, we found that the prevalence of alcohol dependence in individuals with bipolar disorder was 7.6% in men and 3.1% in women, showing an overall increase in the prevalence of alcohol dependence and a reduction in the gender gap. It appears that being affected by bipolar disorder increases the risk of alcohol dependence in women more than men compared to the general population. Given the increasing prevalence of drinking habits among women in the general population in Japan, the need for continued appropriate education and support for alcohol issues has become evident.

Interestingly, the bipolar subtype was also significantly associated with alcohol dependence. Compared to bipolar II disorder, bipolar I disorder has a greater prevalence of alcohol dependence. Bipolar I disorder is defined as more severe manic episodes than bipolar II disorder based on diagnostic criteria (20). Additionally, alcohol consumption by patients with bipolar disorder can increase the risk of manic episodes (29). In our study, mood status was also found to be associated with alcohol dependence. Specifically, a statistically significant association was observed between a manic/hypomanic state or mixed features and alcohol dependence compared to remission overall. However, no significant association was observed for depressive state. According to treatment guidelines, pharmacotherapy for acute mania includes lithium and valproate as first-line mood stabilizers (30). Quetiapine, asenapine, and paliperidone have also been reported to be effective for treating and preventing acute mania (30). For this reason, it appears that individualized pharmacotherapy strategies based on the guidelines with a focus on manic states might reduce the risk of alcohol dependence in patients with bipolar disorder.

As an explanation for the relationship between alcohol dependence and mood disorders, the “self-medication hypothesis” has garnered increasing attention (31). Alcohol, a substance that temporarily relieves anxiety or induces euphoria, is often used as a means of coping with stress for escape purposes (32). It is also suggested that alcohol, due to its sedative effects, may be consumed in an attempt to alleviate mood disturbances caused by excessive euphoria (33). However, alcohol has both physical and psychological dependence effects, which can worsen the prognosis of patients with bipolar disorder. Therefore, evidence suggests that it is essential to maintain remission of bipolar disorder through appropriate pharmacotherapy, which primarily consists of mood stabilizers and antipsychotics (30). It is also important to not overlook alcohol dependence in the treatment of bipolar disorder because social problems may manifest in the context of alcohol problems, leading to secondary mood declines (34). Thus, it is crucial to address alcohol issues from a psychosocial perspective in the treatment of outpatients with bipolar disorder.

The key point is that integrated treatment of alcohol dependence and bipolar disorder contributes to maintaining remission of psychiatric symptoms in patients (35). This is because alcohol dependence is one of the factors that worsens the symptoms of bipolar disorder and leads to poor treatment outcomes (36). Similar to our research findings, alcohol dependence is also known to increase the risk of mood episodes (especially mania and mixed states) in patients with bipolar disorder (37). Interestingly, it has been reported that not only medications specifically for alcohol dependence, such as naltrexone and acamprosate, but also medications for bipolar disorder, such as lithium carbonate and valproic acid, can help improve alcohol-related issues in patients with both conditions (37, 38). Successful treatment of alcohol dependence may reduce the risk of relapse and suicide in patients with bipolar disorder, and an approach combining individualized pharmacotherapy and psychosocial support is considered particularly effective (36).

Previous studies have emphasized that the treatment setting for comorbid cases of bipolar disorder and alcohol dependence primarily focuses on outpatient care (22). However, patients with comorbid bipolar disorder and alcohol dependence are more likely to miss outpatient appointments and have poor medication adherence. Even with treatment, patients with alcohol dependence are reported to have short-term remission rates limited to 20-50% (39, 40). Those who do not receive support have even higher rates of relapse (41). Due to the strong association of alcohol dependence with social isolation (42), achieving recovery requires not only pharmacotherapy and individual psychotherapy but also group psychotherapy facilitated by self-help groups and collaboration involving social workers. Furthermore, since comorbid substance use disorders have been reported to increase the risk of hospitalization for patients with bipolar disorder (43), collaborative care between medical facilities with inpatient capabilities and psychiatric clinics is essential to avoid crisis.

This study revealed a significant association between the presence of psychotic symptoms and alcohol dependence in women with bipolar disorder. Alcohol use disorder has been identified as a vulnerability factor for psychotic symptoms, particularly hallucinations, in individuals with bipolar disorder (44). The comorbidity of psychotic symptoms is associated with greater severity and affects subsequent functional outcomes. Compared with men, women are reported to develop alcohol problems when they consume even small amounts of alcohol, and they are at greater risk for binge drinking, suggesting the need for gender-specific interventions for alcohol dependence (45). Additionally, women have a higher incidence of bipolar disorder during the perinatal period (46). During this time, the comorbidity of alcohol dependence may increase the risk of fetal alcohol spectrum disorder (47) and child abuse (48), potentially causing adverse effects not only for the individual but also for children. Therefore, gender-specific approaches to addressing bipolar disorder and alcohol dependence are important.

This study has several limitations. First, because this was an observational study with a cross-sectional design, causal relationships between factors cannot be determined. In addition, this study included outpatients in Japan but lacked information on inpatients. Thus, excluding inpatient populations who may have more severe comorbidities, limiting the generalizability to all individuals with bipolar disorder. Moreover, this study included only clinics affiliated with the JAPC, which may have introduced selection bias. Patient selection was not random but rather retrospective, which may also have introduced selection bias. The reliance on retrospective data from medical records may introduce bias, as the study lacks control over data accuracy and completeness. Additionally, the tendency toward alcohol dependence in this study was not quantitatively assessed for severity. To establish more robust evidence in the future, it is necessary to stratify the level of alcohol dependence using assessment criteria such as the Addiction Severity Index (49) and to more accurately assess risk factors for comorbid alcohol dependence and bipolar disorder. The study uses binomial logistic regression on a large sample size of participants, but the absence of a validation cohort to cross-check findings could raise concerns about model overfitting. The study also found that other psychiatric comorbidities were significantly associated with alcohol dependency, but the sample size was insufficient for stratified analysis of each psychiatric comorbidities (e.g., attention deficit hyperactivity disorder, panic disorder, personality disorder). Therefore, it was considered necessary to conduct the study on a larger population in order to generate more robust evidence.

## Conclusions

We found that outpatients with bipolar disorder had higher rates of alcohol dependence overall than the general population in Japan, especially among males. In addition, the relationship between substance use disorder and suicidal ideation was stronger in women than in men with bipolar disorder. There was also a strong association between manic states and alcohol dependence in outpatients with bipolar disorder. These results are useful to clinicians because they reinforce real-world clinical evidence for the treatment of bipolar disorder and co-occurring alcohol dependence.

## Data Availability

The raw data supporting the conclusions of this article will be made available by the authors, without undue reservation.
